# Ensemble landmarking of 3D facial surface scans

**DOI:** 10.1038/s41598-017-18294-x

**Published:** 2018-01-08

**Authors:** Markus A. de Jong, Pirro Hysi, Tim Spector, Wiro Niessen, Maarten J. Koudstaal, Eppo B. Wolvius, Manfred Kayser, Stefan Böhringer

**Affiliations:** 1 Department of Oral & Maxillofacial Surgery, Special Dental Care, and Orthodontics, Erasmus MC University Medical Center Rotterdam, Rotterdam, 3015 CE The Netherlands; 2 Department of Medical Statistics and Bioinformatics, Leiden University Medical Center, Leiden, 2333 ZC The Netherlands; 3 Department of Genetic Identification, Erasmus MC University Medical Center Rotterdam, Rotterdam, 3015 CE The Netherlands; 4 Department of Twin Research & Genetic Epidemiology, King’s College London, London, SE1 7EH United Kingdom; 5 Department of Medical Informatics, Erasmus MC University Medical Center Rotterdam, Rotterdam, 3015 CE The Netherlands; 6 Faculty of Applied Sciences, Delft University of Technology, Delft, 2628 CJ The Netherlands

## Abstract

Landmarking of 3D facial surface scans is an important analysis step in medical and biological applications, such as genome-wide association studies (GWAS). Manual landmarking is often employed with considerable cost and rater dependent variability. Landmarking automatically with minimal training is therefore desirable. We apply statistical ensemble methods to improve automated landmarking of 3D facial surface scans. Base landmarking algorithms using features derived from 3D surface scans are combined using either bagging or stacking. A focus is on low training complexity of maximal 40 training samples with template based landmarking algorithms that have proved successful in such applications. Additionally, we use correlations between landmark coordinates by introducing a search strategy guided by principal components (PCs) of training landmarks. We found that bagging has no useful impact, while stacking strongly improves accuracy to an average error of 1.7 mm across all 21 landmarks in this study, a 22% improvement as compared to a previous, comparable algorithm. Heritability estimates in twin pairs also show improvements when using facial distances from landmarks. Ensemble methods allow improvement of automatic, accurate landmarking of 3D facial images with minimal training which is advantageous in large cohort studies for GWAS and when landmarking needs change or data quality varies.

## Introduction

Interest in facial analysis has recently surged in genetic and genome-wide association studies (GWASs), partly due to the availability of large cohorts and partly due to availability of efficient surface scanning^1^. The aim of such studies is to explain phenotypic variation as a first step in understanding the genetic basis of the human face^1–8^. This situation contrasts with facial analysis in clinical genetics in which samples sizes are usually much smaller. In the clinical application, shape differences between groups tend to be large^9–12^ within small cohorts, whereas in population based applications such as GWASs shape differences due to genetic variation are usually small^1–3^. As landmarking structure and input data vary across studies^1,9,13^, and as such require manual retraining of landmarking algorithms, both applications benefit from low training complexity.

Promising results for landmarking algorithm accuracy have been demonstrated by several landmarking approaches so far^14–16^, some heavily depending on heuristics^17,18^. Still, it is unclear whether strengths of individual algorithms are complementary, *i*.*e*. whether they can be combined to generate yet more accurate landmarking data. In previous work, we showed that different data transformations make additional information available to standard wavelet-based methods^15^. However, we noted two drawbacks that we overcome with the present study. Firstly, our previous approach performs unsatisfactorily for landmarks in areas with little structural information such as the forehead or the chin region. Secondly, the choice of transformations that we used as input for our algorithm was not systematic or weighted, leaving open the question of optimality.

To address the first problem, we note that the distribution of landmark positions in the (training) population may provide additional information about the landmarks with little structural information. Such information can be exploited by using principal component analysis (PCA) of the landmark space such as used by active shape models^19^. The second problem poses a model selection problem wherein information from different input data transformations, or features, needs to be weighted and selected for each individual landmark.

In this study, we explore model selection of input features in combination with information from PCA of population coordinates under the constraint of small training samples. We employ the statistical ensemble methods of bagging and stacking to integrate all landmarking information into a single landmarking method. Model selection is performed as an intrinsic feature of the stacking combination technique^20^.

In a broad sense, ensemble methods have been employed in landmarking algorithms before. Elastic bunch graph matching (EBGM)^16^, the method used for most of the base landmarking methods in this and our previous paper, can be viewed as an ensemble method as it integrates a bank of varying wavelet filters into a single matching score per landmark. However, no weighting takes place.

More recently, deep learning technology has been used in the landmarking problem^21^. Deep learners can also be viewed as ensembles, where base learners are repeatedly integrated in each new layer of the network. However, deep learning methodology is not suitable for the smaller training sample sizes we consider here. For example, one study made use of 20,000 training samples^21^. We therefore do not consider deep learners and focus on combination methods suitable for small training samples.

The paper is organized as follows: first, we describe the new landmarking algorithm. In the next sections, we detail several landmarking experiments that are evaluated using either cross-validation or heritability and present the results. We conclude with a discussion.

## Methods

The landmarking algorithm presented here combines a number of base landmarking algorithms into an ensemble. A base landmarking algorithm can be any algorithm that can propose a landmark position given new input data. To abbreviate, we will refer to an individual landmarking algorithm as a *landmarker* in the following. Averages or regression predictions are used to predict the final landmark from landmarks proposed by the base landmarkers. In our implementation, all base landmarkers are template based. A small number (typically 30 to 40) of training images is manually labeled by a rater from which base landmarkers extract templates in the training phase.

As a pre-processing step, 2D projections of the raw 3D surface data are derived. 3D information is retained in a heightmap that corresponds point-wise to a 2D texture, making the transformation one-to-one. A number of features are generated from this combined 2D data that serve as input for the base landmarking algorithms.

All base landmarkers are based on Gabor wavelet responses. Most algorithms target different features and work analogously to the EBGM algorithm with local search strategies. An algorithm with a global search strategy based on principal components (PCs) is added to the ensemble. The choice of base landmarkers is discussed later. The landmark search for the base algorithms is initialized at the population mean.

### Projection and Data preprocessing

Projection of 3D surface data onto a 2D plane works by fitting an ellipsoid to the facial surface data and applying a Mercator map projection that results in a relief map.

**Figure 1:**
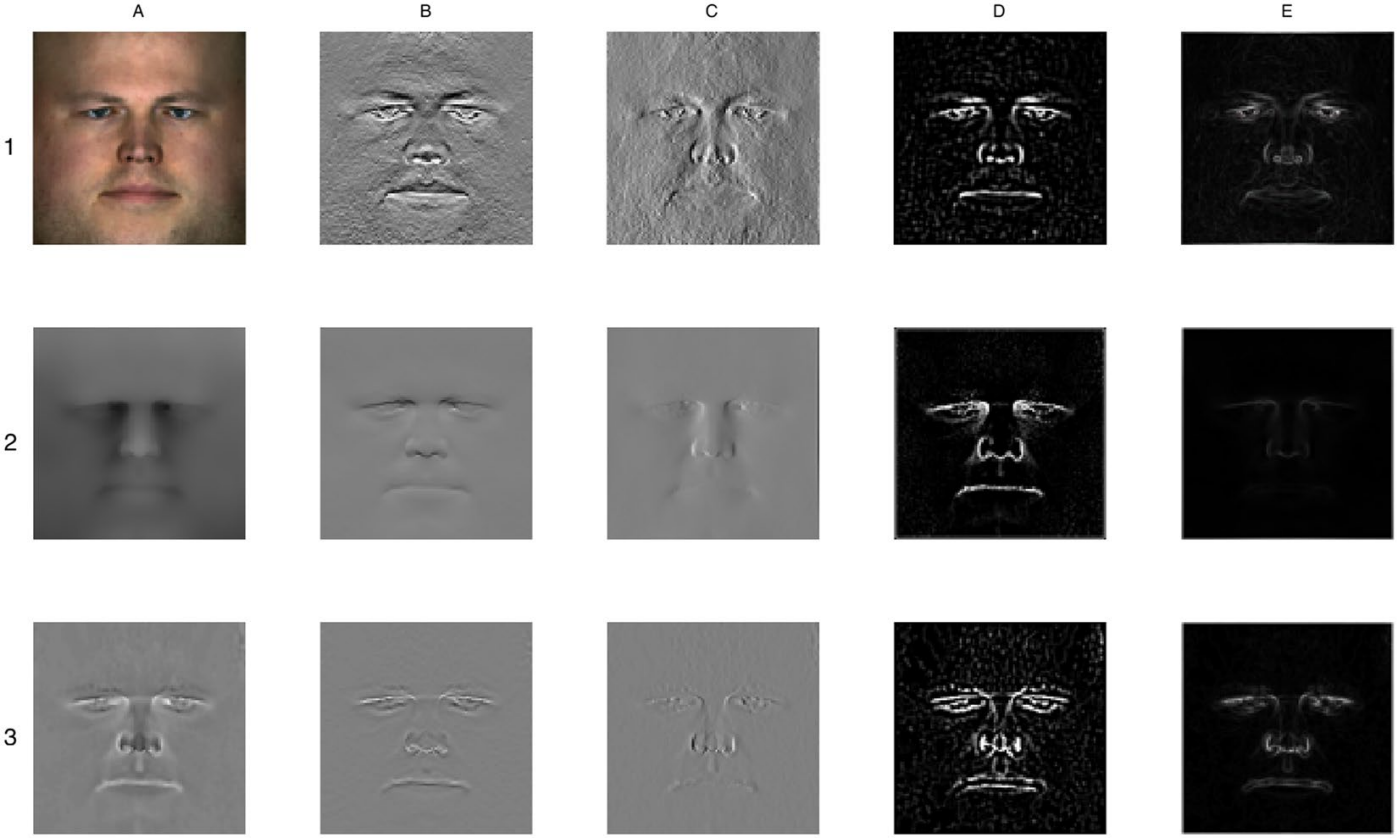
Feature set overview. Main feature displayed in the first column: (1**A**) texture, (2**A**) heightmap. (3**A**) curvature. The remaining columns show edge enhancements of the main features: (**B**) derivative over x-axis, (**C**) derivative over y-axis, (**D**) Laplacian of Gaussian filter, (**E**) Sobel filter. For illustration purposes, the face used in this image is that of author MadJ who was not a participant this study.

The region of interest (ROI) of the frontal face is delimited by a standard sized square placed following the map projection (for an example of the ROI, see Fig. 1(1A)). The size of the 2D features generated from the 3D surface is 200 × 200 pixels (40,000 pixels total).

### Feature set

Three main features are created by using data components that correspond one-to-one per pixel: photographic or *texture* (Fig. 1(1A)), heightmap (Fig. 1(2A)) and curvature (Fig. 1(3A)).

The main texture feature is created directly from the map projection using the original photographic information attached to the map projection^15^.

The main heightmap feature is based on the elevation levels with respect to the ellipsoid that were retained after the map projection^15^.

The third main component, curvature, is newly introduced and derived as follows: first, the curvature per 3D edge of the surface mesh is calculated by taking the mean normal of the first two principal curvatures of the attached triangular surface patches. Secondly, within each patch of the triangulation, curvatures are computed by linear interpolation based on curvatures of the three related edges. Thirdly, curvatures are projected onto 2D using the projection derived above.

To enrich the number of available features, several data transformations that can be described as edge enhancements are applied to these components. These are: vertical and horizontal directional pixel derivatives, a Laplacian of Gaussian (LoG) filter and a newly introduced Sobel filter (Fig. 1, columns B–E). In tests, each of these filters have shown good performances for non-overlapping subsets of landmarks. Any information overlap is expected to be removed through feature selection with ensemble methods. In total, 15 features are generated that form the input of the base landmarking algorithms.

Due to the one-to-one correspondence of pixels between features, training landmarks only have to be placed on a single feature image to be used for the complete set.

### Base landmarking algorithms

Most base landmarking algorithms, or landmarkers, are based on the EBGM algorithm. In the training phase, a set of Gabor wavelets of different sizes and orientations is convoluted with all 15 individual features and the filter responses are extracted at the training landmarks, representing the templates. These responses are stored in a “bunch graph”. In the landmarking phase, the set of Gabor wavelets is applied to a new image to be landmarked. Then, the bunch graph is read for a template search in which responses from the training data are correlated with responses from the new image. The pixel coordinate for which maximum correlation is achieved, serves as the landmark prediction. Details of this procedure are given elsewhere^15,16,22^.

A first set of 15 base landmarkers is based on the individual features above, employing an EBGM algorithm on each. This set is augmented by two additional landmarkers. One base landmarker uses the sum of the wavelet responses of the 15 features for a template search. Another simply averages the final output coordinates of the 15 base landmarkers.

### Principal Components

EBGM performs a local search around the starting position. To exploit correlations between coordinates of different landmarks, we introduce base landmarkers making use of PCA derived information.

For a given set of landmarks, PCA is performed on the landmark coordinates of training samples. The first two principal components (PCs) are used to direct a global search across these landmarks simultaneously.

Specifically, a neighborhood in the PC space is explored in a grid search on the first two PCs across all selected landmarks in the graph and across all features simultaneously, looking for a maximum combined correlation. The grid search is limited to a rectangular neighborhood, the size of which is defined by one standard deviation for that landmark in the training sample.

**Figure 2:**
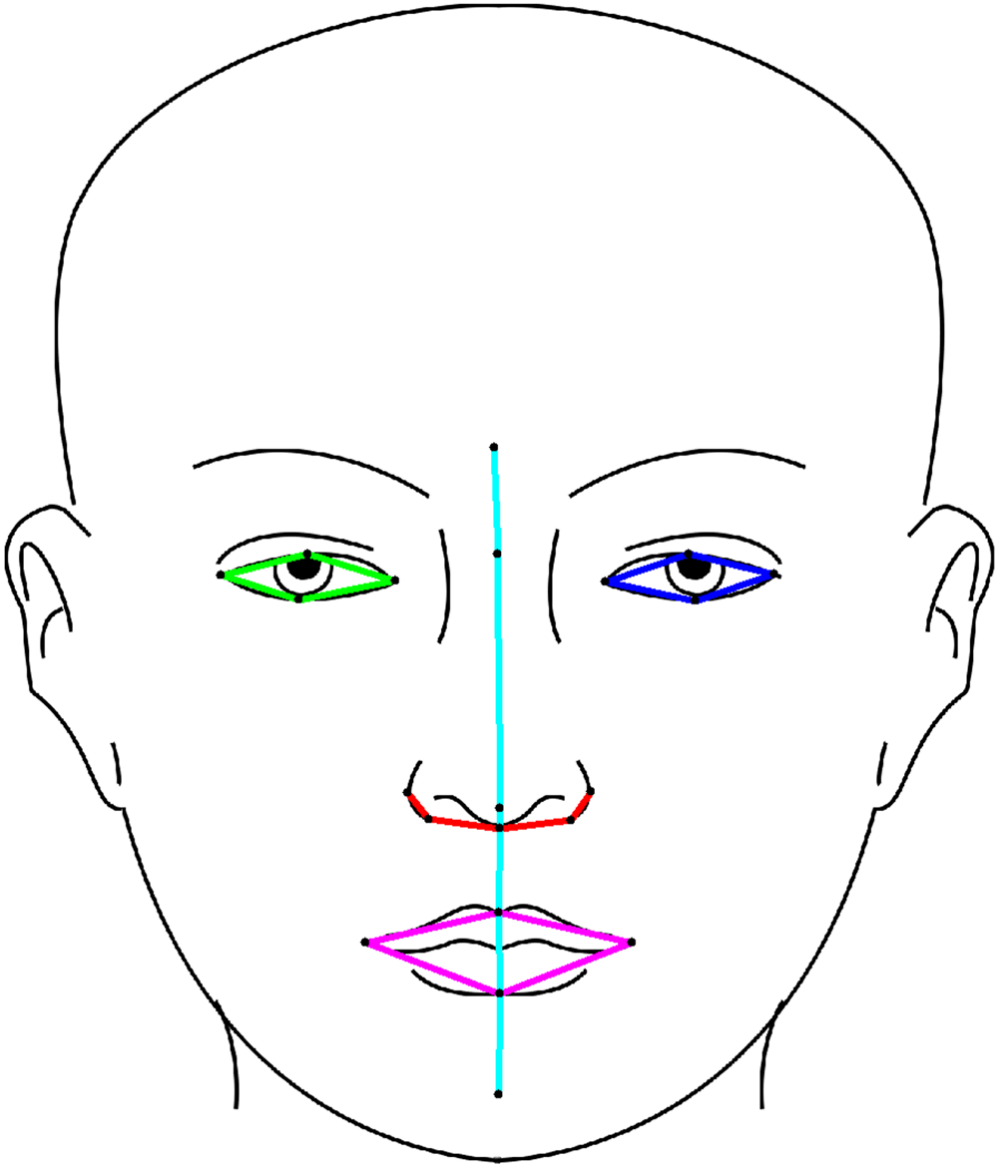
Illustration of the 5 PC sub-groups.

PCA exhibits high variability in loadings for small sample sizes^23^. For this reason, the facial graph is subdivided into five sub-graphs (Fig. 2) to keep the number of variables small (4–6 landmarks) in relation with the sample size (30 or 40 training samples). The choice of sub-graphs is based on expected natural correlation between landmarks and symmetries. Otherwise, no systematic evaluation of possible sub-graphs was performed.

Whenever a sub-graph contains landmarks that overlap with a previously fitted sub-graph (i.e. the cyan group in Fig. 2), the search is additionally penalized by the distance between overlapping landmarks.

### Summary of base landmarkers

To summarize, we consider the following 18 base landmarkers:

- (1–15) The 15 landmarkers applying EBGM on individual features.
- (16) The landmarker based on the sum of the wavelet responses from landmarkers 1–15.
- (17) The mean of the final coordinates from landmarkers 1–15.
- (18) The PC-based landmarker.

### Ensembles

Ensembles are uses to combine base landmarkers into a final landmarking algorithm. We consider two ensemble techniques: bagging, also known as bootstrap aggregating, and stacking, also known as stacked generalization^20^. Bagging has a smoothing property, whereas stacking has model selection properties by means of weighting base landmarkers^20^.

#### Bagging

The idea behind bagging is to create a large number of random sub-samples taken from a data set with replacement, called bags, after which fitting takes place in each of these bags. The final result is an average of the predictions of the individual models, with the intention that the average leads to a more stable predictor with less overfit than a single model fitted to the data would have.

In the present case, bags are created from the training data (30 facial scans) using 15 features. For each of these bags, base landmarkers are fitted that extract templates from the bags. Predicted landmark coordinates are averaged across the bags to give the final landmark position. Details of the bagging algorithm as used in this implementation are given in Algorithm 1.

**Algorithm 1:**
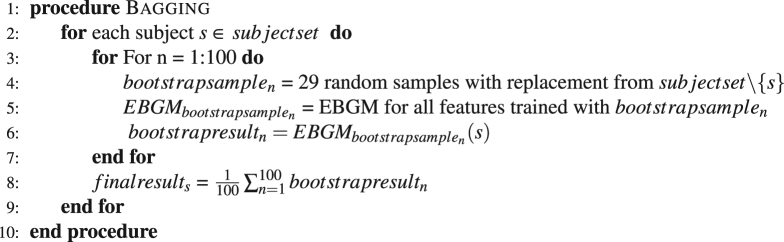
30 item leave-one-out Bagging algorithm.

#### Stacking

In stacking, predictions from multiple low-level learning algorithms are used as input for a final combining top-level learning algorithm^20^. Stacking can be viewed as a feature selection procedure, as the final combiner typically weights the low-level algorithms. In our algorithm, the base landmarkers listed above are used for the low-level learning step. For the combination step, a least squares linear regression was applied. The three best predicting base landmarkers were selected according to the regression coefficients to create the final top-level predictor with these relative weights. Details of the stacking algorithm as used in this implementation are given in Algorithm 2.

**Algorithm 2:**
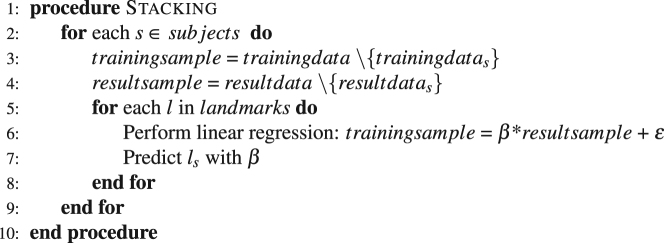
40 item stacking algorithm.

### Heritability

Apart from cross-validation, we also used heritability to evaluate landmarking performance. Heritability is defined as the percentage of variation in a trait explained by genetic effects. Heritability can be estimated from families using a mixed effect model for which the variance of the random effect represents genetic effects and can be compared with residual variation^24^. We used twin data from the TwinsUK cohort for these analyses which included 37 monozygotic and 163 dizygotic twins. We estimated narrow sense heritability which assumes additive genetic effects for a number of features derived from landmark coordinates. To this end, we used a triangulation of the symmetrized mean graph to define a triangle structure. Then, coordinates were subjected to a Procrustes analysis using R package *shapes* and all distances between pairs of landmarks and all angles and areas of triangles were calculated for each of the samples. Heritabilities were calculated for each of these features as well as for landmark coordinates.

Heritabilities were visualized using importance plots which summarize heritabilities across features by computing a weighted average of the heritabilities for each point in the image. The weighting is linear in both the size of the individual heritability and the inverse distance of the center of the feature with the current point. Details of this procedure are given elsewhere^25,26^.

## Experiments

The study and its experiments were conducted throughout 2016. All methods were performed in accordance with Erasmus MC guidelines and regulations according to which this study was not subject to evaluation by the medical ethical committee (http://www.ccmo.nl/en/non-wmo-research).

### Data set

The data set used in the performance assessment of the presented algorithm is a random selection of 40 non-twin subjects from the *TwinsUK* cohort. The TwinsUK cohort consists of exclusive European descent.

The cohort consists of volunteers drawn from the general British population, unaware of any 3D studies scientific interests at the time of enrollment and gave fully informed consent under a protocol reviewed by the St. Thomas’ Hospital Local Research Ethics Committee. Reference: PMID 23088889.

The *TwinsUK* dataset has models with ca. 1.5 × 10^5^ points and textures with resolution of ca. 2,000 × 1,000 pixels. The data set was acquired with *3dMDface* photogrammetric systems^27^.

#### Data availability

Due to privacy restrictions, raw data (i.e. facial 3D surface scans) cannot be made available for download. Subject to evaluation of a research proposal, the TwinsUK data set is made available by co-authors PH and TS.

### Accuracy estimation

Cross-validations were performed to evaluate accuracy for a set of 21 landmarks. The different landmarkers were tested in leave-one-out experiments in which the ground truth consisted of a single manual labeling of the entire data set.

Additionally, we estimated heritability on the whole data set which can be done without knowing the ground truth.

### Feature Set and Principal Components

All individual features and the PC-based predictions were all tested with a 40-item leave-on-out setup.

### Bagging

Bagging was tested with a 30-item leave-one-out setup. Due to the large amount of iterations that were required (60,000), the experiment was performed on a computer cluster.

### Stacking

Stacking of the base landmarkers was tested with a 40-item leave-one-out setup that included the complete feature set and PC predictions.

## Results

### Cross-validation

Results in this paper are compared to previous results, called the benchmark, as given by a previous version of our algorithm^15^ that did not make use of ensemble learning. This earlier algorithm has been shown to outperform an active shape model based landmarking approach for most landmarks^15^.

**Figure 3:**
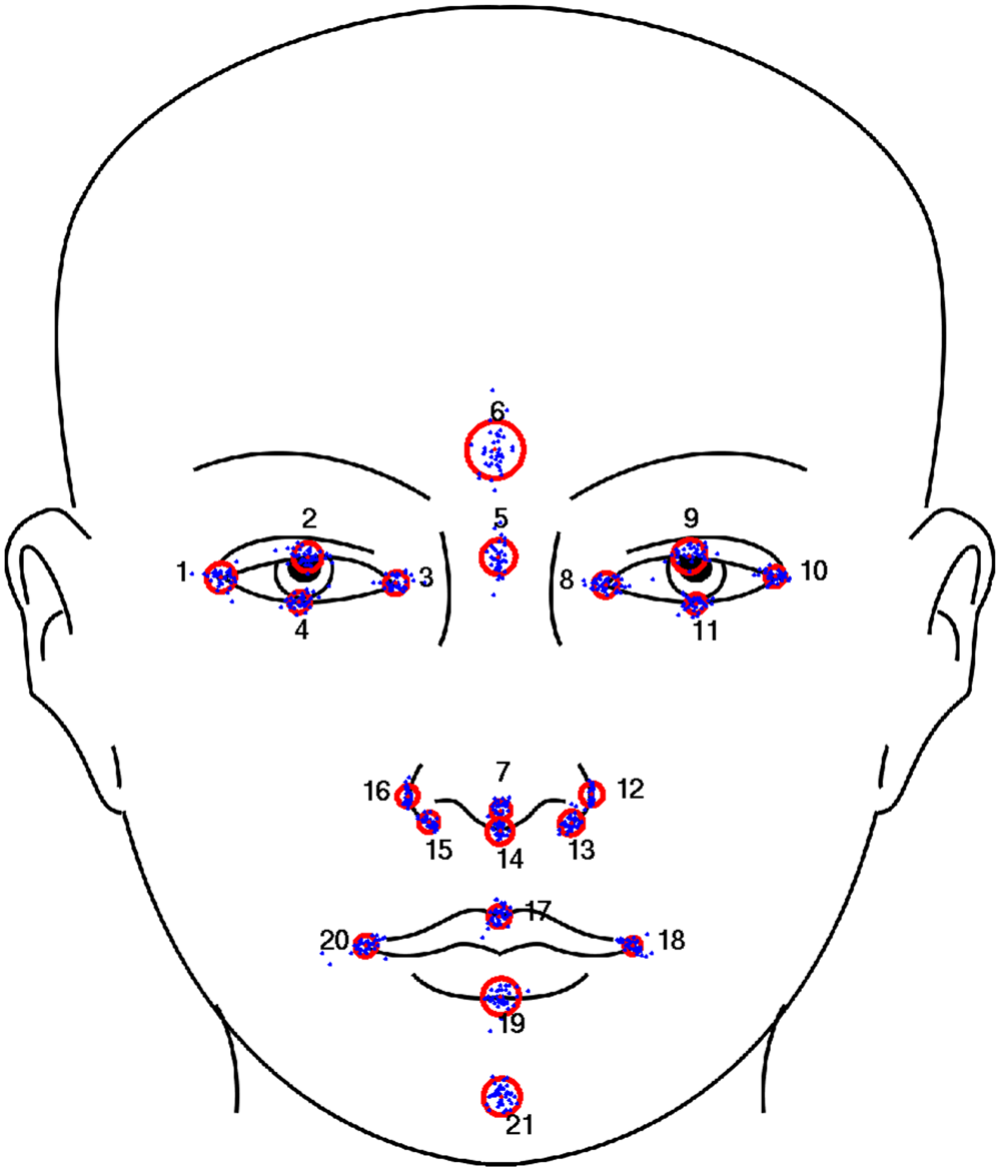
Stacking final results.  Relative landmark result spread, all 40 final leave-one-out landmark results are plotted over each other.  Mean distance to the training landmarks.

Table 1 shows the results for each of the 15 base landmarkers obtained by EBGM from the respective features. Table 2 shows results for ensemble methods together with benchmark results from our previous algorithm. Both tables report Euclidean distance to the ground truth (training data) in mm. The final, stacked results are visualized in Fig. 3.

**Table 1:** Automatic landmarking results for 15 base landmarkers.

Landmark	Texture					Heightmap					Curvature				
	*Ori*	*Dx*	*Dy*	*LoG*	*Sob*	*Ori*	*Dx*	*Dy*	*LoG*	*Sob*	*Ori*	*Dx*	*Dy*	*LoG*	*Sob*
1	*4*.*5*	*7*.*8*	*5*.*9*	*7*.*5*	3.7	*4*.*4*	*5*.*0*	*8*.*0*	*8*.*5*	*4*.*9*	*5*.*9*	4.0	3.2	3.6	*4*.*5*
2	3.6	*7*.*6*	3.9	*6*.*9*	*5*.*0*	3.9	*4*.*4*	*4*.*7*	*9*.*2*	3.2	3.9	3.5	2.6	3.7	3.3
3	3.8	*5*.*4*	3.0	3.5	3.2	3.0	2.6	*6*.*7*	*5*.*2*	3.5	3.0	2.4	2.4	3.3	2.6
4	3.7	*5*.*6*	*4*.*8*	*6*.*7*	3.7	2.8	2.0	*4*.*6*	*7*.*0*	3.6	*4*.*6*	*4*.*2*	1.9	2.5	3.6
5	*4*.*0*	2.3	*4*.*3*	*5*.*4*	*4*.*3*	*5*.*4*	3.6	*6*.*0*	*7*.*5*	*4*.*8*	*6*.*7*	3.8	*6*.*2*	*4*.*4*	*6*.*1*
6	*6*.*2*	*8*.*0*	*6*.*5*	*5*.*8*	*6*.*2*	*7*.*3*	*6*.*3*	*7*.*0*	*8*.*1*	*6*.*6*	*7*.*0*	*6*.*7*	*6*.*1*	*6*.*6*	*7*.*5*
7	2.3	*4*.*9*	3.0	3.1	2.6	2.2	2.0	*5*.*1*	*6*.*8*	2.6	2.0	2.3	1.9	2.0	2.3
8	3.2	*4*.*8*	*4*.*2*	*8*.*6*	3.5	3.2	3.0	*5*.*8*	*6*.*4*	*4*.*1*	2.9	2.9	*4*.*5*	2.5	2.6
9	*4*.*4*	*4*.*3*	3.5	*8*.*1*	*4*.*2*	*4*.*7*	4.0	*5*.*4*	*8*.*3*	3.8	*4*.*9*	3.6	3.1	3.6	3.4
10	3.9	*5*.*0*	*5*.*7*	*7*.*8*	*4*.*2*	2.9	3.0	*6*.*3*	*7*.*9*	*4*.*9*	*6*.*7*	2.6	2.2	2.4	2.2
11	3.2	4.0	3.8	*5*.*2*	3.9	2.4	2.2	3.6	*6*.*0*	3.2	2.8	3.0	2.4	2.9	2.2
12	1.9	*6*.*2*	2.6	2.0	2.3	3.0	2.8	*5*.*6*	2.1	2.0	2.1	1.9	2.9	2.9	2.6
13	2.3	2.5	*4*.*5*	2.7	2.1	2.7	2.0	*6*.*4*	2.3	2.1	2.0	2.1	2.2	2.3	2.3
14	2.2	2.3	2.4	3.9	2.4	3.3	3.2	2.8	3.9	3.3	2.3	2.2	2.3	2.7	2.3
15	2.0	2.5	3.6	2.3	2.1	1.8	1.8	3.9	1.9	1.8	2.0	2.1	1.9	1.8	1.9
16	1.9	2.6	2.9	2.3	2.1	2.1	2.4	*4*.*7*	2.3	2.1	2.0	2.3	2.2	2.3	2.0
17	3.9	*6*.*6*	3.3	*5*.*4*	2.5	*6*.*0*	*7*.*9*	*4*.*3*	*8*.*7*	*4*.*1*	3.1	2.5	3.0	3.5	*4*.*7*
18	*4*.*4*	*14*.*4*	*6*.*9*	*14*.*6*	*5*.*0*	3.8	3.4	*17*.*8*	*16*.*7*	*4*.*2*	*10*.*7*	*4*.*0*	1.8	1.8	3.1
19	*4*.*3*	*9*.*6*	*8*.*1*	*12*.*5*	3.7	*6*.*4*	*5*.*5*	*8*.*7*	*12*.*2*	3.6	*6*.*5*	*4*.*7*	*6*.*3*	*4*.*6*	*8*.*7*
20	*4*.*2*	*14*.*2*	*8*.*6*	*12*.*3*	*4*.*9*	3.0	*4*.*5*	*15*.*3*	*17*.*4*	*6*.*0*	*9*.*1*	3.0	*4*.*5*	2.6	*5*.*0*
21	*8*.*9*	*8*.*2*	*11*.*1*	*12*.*8*	*11*.*8*	*12*.*0*	*7*.*5*	*11*.*1*	*14*.*4*	*4*.*2*	*12*.*7*	3.3	*8*.*5*	*6*.*6*	11.3
mean	3.8	6.1	4.9	6.6	4.0	4.1	3.8	6.8	7.7	3.7	4.9	3.2	3.4	3.3	4.0
sd	1.6	3.5	2.3	3.8	2.1	2.3	1.8	3.7	4.4	1.3	3.1	1.1	1.9	1.4	2.5

Results are reported in Euclidean distance to manual training data in mm, split by main feature (texture, heightmap, curvature) and sub-feature: Ori = no filter, Dx = derivative over x-axis, Dy = derivative over y-axis, LoG = Laplacian of Gaussian filter, Sob = Sobel filter. Distances < 2 mm are underlined, distances > 4 mm are in *italics*.

**Table 2:** Ensemble landmarking and PC results.

Landmark	[Benchmark]		SoWR 15	Mean 15	PC	[Bagging]	[Stacking]	
1	2.4	(3.1)	2.4	3.1	*4*.*4*	3.7	1.8	(1.3)
2	1.7	(0.8)	2.4	2.8	2.8	2.4	2.1	(1.4)
3	1.8	(1.0)	1.9	1.9	2.6	1.8	1.6	(1.0)
4	2.3	(1.7)	1.7	2.1	2.7	1.4	1.5	(0.8)
5	2.4	(1.9)	2.9	2.4	2.8	2.2	2.0	(1.6)
6	6.5	(4.3)	*4*.*7*	3.3	*4*.*9*	*5*.*8*	3.0	(2.0)
7	2.1	(1.3)	1.7	1.9	*14*.*5*	3.0	1.4	(0.6)
8	1.5	(1.3)	2.0	2.2	3.2	1.5	1.9	(1.0)
9	1.8	(1.2)	2.7	2.7	*4*.*4*	2.8	2.3	(1.9)
10	2.2	(1.2)	1.7	2.4	3.9	3.0	1.6	(0.9)
11	1.7	(1.9)	1.6	1.6	3.1	1.6	1.3	(0.8)
12	1.1	(0.7)	1.9	1.7	*12*.*2*	1.6	1.3	(0.8)
13	1.4	(0.9)	2.0	1.6	*13*.*1*	1.5	1.5	(0.7)
14	1.2	(0.6)	2.2	1.7	*10*.*5*	2.2	1.5	(0.9)
15	1.5	(0.8)	1.6	1.5	*13*.*5*	1.5	1.3	(0.7)
16	1.2	(0.7)	1.7	1.9	*14*.*1*	2.0	1.7	(1.0)
17	1.2	(2.3)	1.8	2.9	*14*.*3*	2.6	1.5	(0.9)
18	2.0	(1.4)	1.4	*4*.*4*	*18*.*3*	2.8	1.4	(0.9)
19	2.5	(3.4)	3.1	3.4	*15*.*8*	3.6	2.1	(2.3)
20	1.9	(3.4)	1.8	*4*.*1*	*18*.*4*	3.0	1.8	(1.7)
21	3.3	(5.4)	3.1	*6*.*1*	2.1	*10*.*0*	2.0	(1.2)
mn	2.1		2.2	2.6	*8*.*6*	2.9	1.7	
sd	1.3		0.8	1.1	6.0	1.9	0.4	

Results are reported in Euclidean distance to manual training data in mm. *Benchmark* represent results from the previous version of our algorithm^15^. Clarification of terms: *SoWR 15* = based on intermediate Summation of Wavelet Responses of 15 landmarkers. *Mean 15* = mean of final coordinates of 15 landmarkers. *PC* = results obtained by our principal component method. Distances <2 mm are underlined, distances >4 mm are in *italics*. Standard deviations are shown in parentheses.

As concluded from our previous algorithm, Table 1 shows that the individual features are able to provide unique information for specific landmarks, implied by small distances for those landmarks.

When studying the results of the newly introduced main curvature feature, it can be seen this feature set improves results for many landmarks, especially 7 (nose tip) and 18 (left mouth corner).

The newly introduced Sobel filter sub-feature, shown in the same table in the leftmost column of each main feature, shows good results for landmark 13 (right nose corner) in the texture feature subset and landmark 12 (right nose outer edge) in the heightmap feature subset. Even though mean distance does not decrease much for those subsets, the Sobel filter contributes to coordinate stability by lowering standard deviations. Their symmetric landmark partners (landmark 16 for 12 and landmark 15 for 13) also perform well but are still outperformed by other features.

The PC results are shown in Table 2 and are compared with our benchmark, results from our previous algorithm^15^. PC-based landmarker successfully improved results for difficult landmarks on the forehead (6) and chin (21), reducing distances from 6.5 mm and 3.1 mm to 3 mm and 2.1 mm respectively. Whilst PC application was especially focused on the forehead and chin, better results should also be attainable for the nose and mouth.

The bagging experiment did not improve results. This is most likely caused by the fact that pertubations to the data such as biological variation, the measure procedure, and labeling errors were roughly comparable across training samples and bagging could not smooth out any outliers due to atypical training samples. Besides these disappointing results, the large computational cost suggests limited use of bagging in landmarking.

Using the stacking algorithm, a significant mean improvement of 0.4 mm across all 21 landmarks was achieved in comparison with our benchmark (2.1 mm vs 1.7 mm). A closer closer comparison shows better performance in all landmarks except 2, 8, 9, 12, 13, 14, 16 and 17. Overall, the stacking method algorithm is able to successfully optimize feature selection and is able to reduce distances. Furthermore, standard deviations are greatly reduced, leading to more reliable and stable landmarks.

### Heritability

Heritability estimates for the extracted features are shown in Table 3 for the five most heritable features in each category. Heritabilities for all features are given as supplementary information. The highest observed heritability was 87% for the area of the triangle defined by landmarks 10, 12, and 18. The best distance had heritability of 72%. Angles and coordinates had best heritabilities of 69% and 64%, respectively.

**Table 3:** Heritabilities of geometric features.

Feature	*β*_0_	*β*_age_	*σ*_1_	*σ*_2_	h^2^
**Coordinates**					
c_12_x	−16.31	−0.01	0.76	1.01	0.64
c_1_x	−43.16	−0.00	1.05	1.31	0.61
c_18_x	−24.47	−0.01	1.36	1.44	0.53
c_3_x	−17.35	−0.02	0.93	0.95	0.51
c_13_x	−12.17	−0.02	0.80	0.74	0.46
**Distances**					
d_3_13	50.62	0.05	1.10	1.78	0.72
d_3_18	73.62	0.09	1.82	2.80	0.70
d_1_8	60.75	0.02	1.51	2.23	0.69
d_1_18	91.10	0.07	1.89	2.78	0.69
d_4_16	32.72	0.04	1.49	2.15	0.68
**Areas**					
ar_18_12_10	71.15	10.58	38.39	100.00	0.87
ar_8_7_12	527.87	0.60	40.85	50.10	0.60
ar_8_7_5	455.48	1.60	41.90	49.52	0.58
ar_14_13_7	122.52	0.35	14.82	13.30	0.45
ar_13_18_12	95.57	0.23	18.39	14.45	0.38
**Angles**					
an_18_12_10_b	2.02	0.00	0.06	0.09	0.69
an_18_12_10_a	0.69	−0.00	0.04	0.05	0.59
an_13_17_18_b	1.02	0.00	0.08	0.09	0.55
an_19_17_18_b	1.13	0.00	0.07	0.08	0.55
an_18_12_10_c	0.43	0.00	0.04	0.04	0.50

*β*_0_, *β*_age_ represent fixed effects of the model, *σ*_1_, *σ*_2_ are variances of the residual error and random effect, respectively.

**Figure 4:**
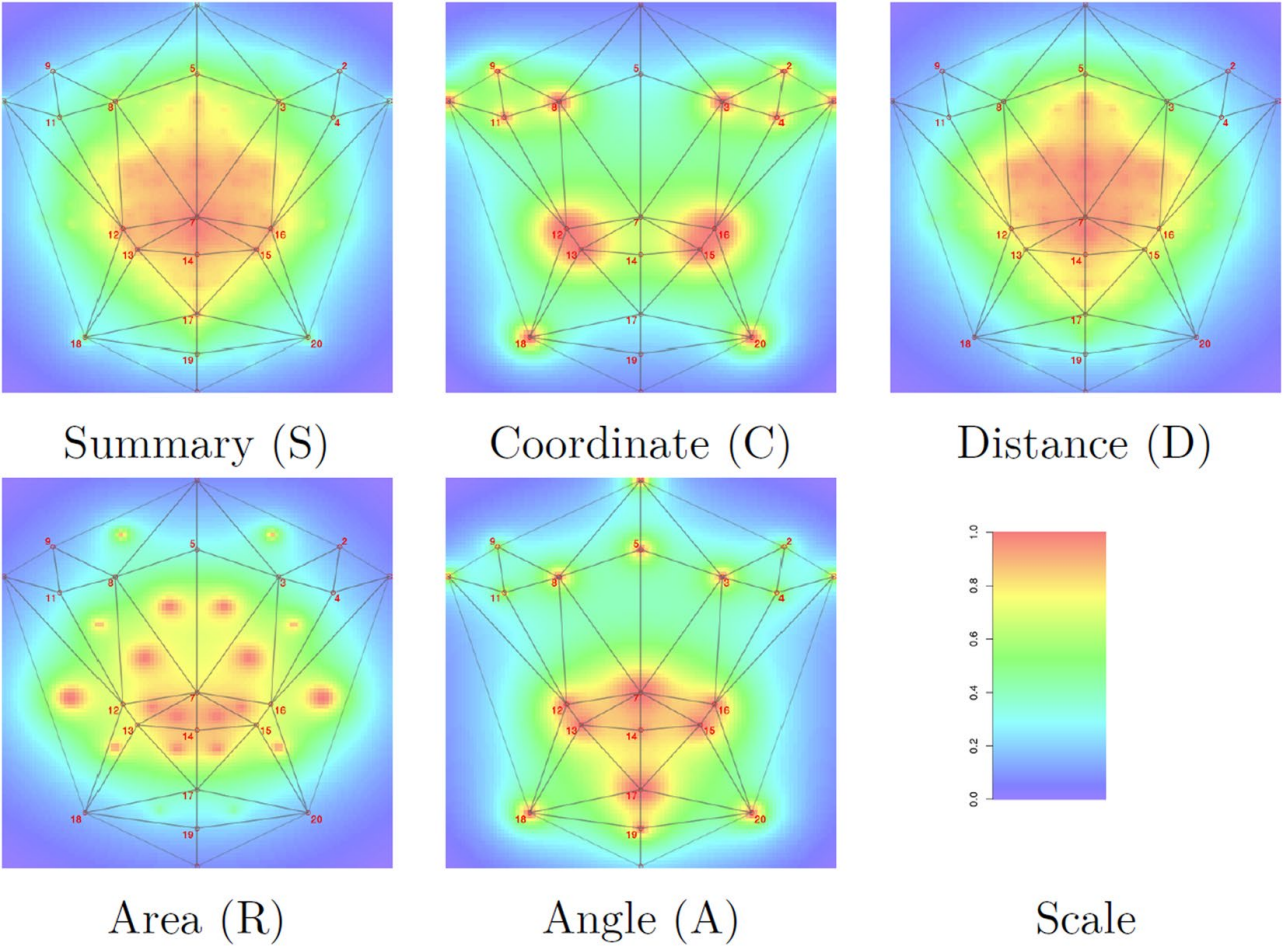
Importance plots of heritabilities of coordinates (C), distances (D), areas (R), angles (A), and a summary (S). Each color scale represents heritabilities which are re-scaled between 0 (blue) and the maximal heritability (red) for the respective feature.

Graphical summaries of heritabilities by means of importance plots are given in Fig. 4. By comparing the overall summary 6 (S) with components C, D, R, and A it is apparent that distances contribute most to overall heritability. Heritabilities for all features except the raw coordinates are concentrated in the central area of the face. To analyze similarities within related individuals in the periphery, it is arguably better to work with the raw coordinates as indicated by Fig. 4(C).

When comparing these results with benchmark results, the best heritability for distances improved from 66% to 72%. In general, heritabilities improved by ~5% when comparing the sorted lists for distances although the distances were not the same.

## Discussion

In this paper, we evaluated ensemble methods to integrate information from several landmarkers (or base landmarking algorithms) in order to improve landmarking accuracy. This approach was motivated by experiences in previous landmarking efforts^15^. By experimenting with different selections of features, it became apparent that features contribute only to a subset of landmarks. Additionally, some landmarks were poorly placed as revealed by inter-rater disagreement which was sometimes caused by atypical training samples. Ensemble methods can address both of these problems. Stacking can downweigh features that are less relevant for a particular landmark and bagging can limit the influence of single training instances by smoothing predictions across bags.

Our results indicate that the composition of the training sample only has a small impact on labeling accuracy as bagging did not improve landmarking accuracy (Table 2). Moreover, this result justifies the use of small training samples as landmarking seems to be robust against changes in training example composition, as bagging contributes this type of variability into the landmarking algorithm. The stacking algorithm resulted in the overall best landmarking accuracy and performed best for almost all individual landmarks (Table 2). Any declines in accuracy for stacking in comparison with the benchmark can most likely be attributed to differences in methodology between both algorithms and 2D to 3D coordinate conversion. Nevertheless, the stacking experiment confirms that contributions of base landmarkers are indeed landmark specific and that a weighted combination can take advantage of this fact.

Symmetric landmarks agree within 0.4 mm of accuracy for stacking, and usually within 0.2 mm. Potential explanations for this symmetrical disagreement are asymmetries in the data, inaccuracies in preprocessing (ROI selection, projection), or random fluctuations due to non-deterministic steps in the algorithm. These comparisons give a sense of the influence of these factors on labeling accuracy and they are roughly an order of magnitude smaller than the accuracies themselves.

In this work, we added new features to the previous algorithm: curvature as main feature and Sobel filter as sub-feature. All of these features did contribute to improve landmarking accuracy for subsets of landmarks. The base landmarker based on a PC-guided search did improve landmarking accuracy for landmarks with little structural information by borrowing information from correlated landmarks. The stacking approach ensures that PC information is used for the appropriate landmarks. It therefore seems a sensible strategy to further enrich the number of available features to improve landmarking accuracy. On the other hand, the explicit need to define features is a disadvantage of our algorithm. Some features do not perform well for any landmark (e.g. Laplacian of Gaussian of the texture) and adding features that are too noisy will most likely decrease landmarking accuracy, despite stacking.

Deep learning offers an interesting alternative by working on raw data directly, thereby circumventing the need to specify features a-priori^21^. A disadvantage of deep learning approaches, however, is the need for big training samples. Up to a thousand-fold increase would be required in comparison to what we use in our current algorithm^21^. This contradicts with our aim to enable fast training of the landmarking algorithms, either for new data sets or for different sets of landmarks. A possible compromise could be to provide a limited number of features and add a network with a smaller number of layers than are used for deep networks trained on big sample sizes. We so far have focused on EBGM based base landmarkers as these can cope with small sample sizes. Using transformations learned from deep learning algorithms - an approach coined transfer learning^28^ - could be a more flexible and generic than our current algorithm and would also retain the advantage of requiring small training samples. Such an approach could be more flexible and generic than our current algorithm and would also retain the advantage of requiring small training samples. It is our intention to investigate such possibilities in future research.

Potentially, large data sets might become available in the near future through consumer grade scanning devices and from social media resources. For such data, low training complexity might be less important. However, we believe that in research settings where data privacy is an important issue and data sets are often older, data specific methods with easy re-training will remain important in the future.

Heritability is an important aspect for genetic analyses. It is more likely to find genetic associations for highly heritable traits than for lesser heritable ones. Several of the estimated heritabilities range between 70% and 80%, values that are also seen in studies using manual landmarks^29^, although it is difficult to compare heritabilities across studies. We mainly use heritability as a benchmark that measures landmarking accuracy. Landmarking errors due to the algorithm contribute to residual variance of a measurement and thereby diminish heritability estimates. In general, estimated heritabilities improved in comparsion with our previous iteration^15^. Distances were the most heritable traits in general and heritability was concentrated in the mid-face, which is a plausible finding. We believe that heritability is a valuable measure for landmarking accuracy when data is available that allows its estimation.

In this study, we present an improved landmarking algorithm for the human face that is based on ensembles and can incorporate an increasing number of features. Selection in the ensemble formation ensures that for a given landmark only useful information is gathered from base landmarkers which in turn make use of specific features. This result is achieved with a low training complexity of 30 to 40 training samples. We were also able to tackle the problem of landmarks with little structural information by using a PC-guided search. Overall we achieved an average accuracy of 1.7 mm, a 22% improvement over our previous algorithm. Furthermore, in comparison with another automated landmarking method with a comparable landmark set^18^, our algorithm shows better overall performance (2.6 mm vs. 1.7 mm for us). This positive comparison also holds when inspecting their best-performing individual landmarks: landmark 7 (tip of the nose) (1.6 mm vs. 1.4 mm for us), and landmark 13 (1.6 mm vs. 1.5 mm for us). Our results show that facial features can be extracted efficiently for large cohorts both in terms of time and cost and thereby enable research on facial morphology in such samples. This includes questions with respect to genetic mechanisms such as pursued in genome wide association studies (GWASs) and medical questions about normal variation, asymmetry, and classification.

## Electronic supplementary material

Supplementary information: Heritabilities for all features
