# Supplementary material for: Ensemble landmarking of 3D facial surface scans
Source: Sci Rep. 2018 Jan 8;8:12. doi: 10.1038/s41598-017-18294-x (PMC5758814; doi:10.1038/s41598-017-18294-x)
Supplement: Supplementary file 1 — Supplementary information: Heritabilities for all features [file 41598_2017_18294_MOESM1_ESM.pdf]

Ensemble landmarking of 3D facial surface scans - Supplementary information: Heritabilities for all features

Markus A. de Jong[1,2,3], Pirro Hysi[4], Tim Spector[4], Wiro Niessen[5,6], Maarten J. Koudstaal[1], Eppo B. Wolvius[1], Manfred Kayser[3], and Stefan Böhringer[2,*]

[1]Department of Oral & Maxillofacial Surgery, Special Dental Care, and Orthodontics, Erasmus MC University Medical Center Rotterdam, Rotterdam, The Netherlands
[2]Department of Medical Statistics and Bioinformatics, Leiden University Medical Center, Leiden, The Netherlands
[3]Department of Genetic Identification, Erasmus MC University Medical Center Rotterdam, Rotterdam, The Netherlands
[4]Department of Twin Research, King's College London, London, United Kingdom
[5]Department of Medical Informatics, Erasmus MC University Medical Center Rotterdam, Rotterdam, The Netherlands
[6]Faculty of Applied Sciences, Delft University of Technology, Delft, The Netherlands
[*]correspondence@s-boehringer.org

ABSTRACT

Supplementary information: Heritabilities for all features

Supplementary information: Heritabilities for all features

Table 1. Heritabilities for all features. *age* effect of age, σ_1 residual error, σ_2 random effects component. h^2 heritability estimate. c_Z_x, c_Z_y denote x and y coordinates of landmark Z, d_X_Y denotes the Euclidean distance between landmarks X and Y, $ar\_X\_Y\_Z$ denotes the area defined by the triangle with vertices X, Y, Z, and $an\_X\_Y\_Z\_T$ is the angle of triangle X, Y, Z at vertex T with a, b, c corresponding to vertices X, Y, Z, respectively.

Response	intercept	age	σ_1	σ_2	h^2
c_1_x	-43,16	0,00	1,05	1,31	0,61
c_1_y	-0,41	0,00	1,13	0,04	0,00
c_1_z	-0,95	-0,01	1,34	0,50	0,12
c_2_x	-32,76	0,01	1,34	1,17	0,43
c_2_y	-0,73	0,01	1,14	0,02	0,00
c_2_z	-0,41	0,00	1,22	0,33	0,07
c_3_x	-17,35	-0,02	0,93	0,95	0,51
c_3_y	-0,61	0,00	0,59	0,27	0,18
c_3_z	-0,36	0,01	0,61	0,35	0,25
c_4_x	-30,47	0,00	1,18	1,06	0,45
c_4_y	-0,59	0,00	0,86	0,21	0,05
c_4_z	-0,34	0,00	0,91	0,09	0,01
c_5_x	6,19	-0,10	0,19	0,86	0,95
c_5_y	6,19	-0,10	0,19	0,86	0,95
c_5_z	6,19	-0,10	0,19	0,86	0,95
c_6_x	6,19	-0,10	0,19	0,86	0,95
c_6_y	6,19	-0,10	0,19	0,86	0,95
c_6_z	6,19	-0,10	0,19	0,86	0,95
c_7_x	6,19	-0,10	0,19	0,86	0,95
c_7_y	6,19	-0,10	0,19	0,86	0,95
c_7_z	6,19	-0,10	0,19	0,86	0,95
c_12_x	-16,31	-0,01	0,76	1,01	0,64
c_12_y	0,13	-0,01	0,78	0,51	0,30
c_12_z	0,69	-0,03	1,39	1,28	0,46
c_13_x	-12,17	-0,02	0,80	0,74	0,46
c_13_y	0,30	-0,01	1,10	0,85	0,37
c_13_z	-0,71	0,00	1,69	0,73	0,16
c_14_x	6,19	-0,10	0,19	0,86	0,95
c_14_y	6,19	-0,10	0,19	0,86	0,95
c_14_z	6,19	-0,10	0,19	0,86	0,95
c_17_x	6,19	-0,10	0,19	0,86	0,95
c_17_y	6,19	-0,10	0,19	0,86	0,95
c_17_z	6,19	-0,10	0,19	0,86	0,95
c_18_x	-24,47	-0,01	1,36	1,44	0,53
c_18_y	-0,53	0,00	1,33	0,52	0,13
c_18_z	0,31	-0,01	1,28	0,83	0,29
c_19_x	6,19	-0,10	0,19	0,86	0,95
c_19_y	6,19	-0,10	0,19	0,86	0,95
c_19_z	6,19	-0,10	0,19	0,86	0,95
c_21_x	6,19	-0,10	0,19	0,86	0,95
c_21_y	6,19	-0,10	0,19	0,86	0,95
c_21_z	6,19	-0,10	0,19	0,86	0,95
d_1_2	17,93	-0,01	1,40	0,71	0,20

Continued on next page

Response	intercept	age	σ_1	σ_2	h^2
d_1_3	26,43	-0,02	1,44	0,56	0,13
d_1_4	15,69	-0,01	1,44	0,55	0,13
d_1_5	48,37	-0,01	1,47	1,29	0,43
d_1_6	55,09	0,00	1,45	1,64	0,56
d_1_7	66,93	0,01	2,26	2,50	0,55
d_1_8	60,75	0,02	1,51	2,23	0,69
d_1_9	77,30	-0,01	2,16	2,33	0,54
d_1_11	74,18	0,00	1,82	2,37	0,63
d_1_12	69,58	0,03	1,64	2,09	0,62
d_1_13	70,18	0,03	1,49	2,03	0,65
d_1_14	64,28	0,02	1,84	2,17	0,58
d_1_15	53,07	0,01	1,59	1,77	0,55
d_1_16	45,18	0,03	1,61	2,21	0,65
d_1_17	72,55	0,07	2,17	2,63	0,59
d_1_18	91,10	0,07	1,89	2,78	0,69
d_1_19	80,36	0,07	2,52	2,57	0,51
d_1_20	63,91	0,09	2,15	3,05	0,67
d_1_21	90,88	0,03	3,10	2,48	0,39
d_2_3	19,29	-0,04	1,57	0,98	0,28
d_2_4	14,68	-0,02	1,65	0,05	0,00
d_2_5	34,47	-0,01	1,31	1,22	0,47
d_2_6	38,61	0,00	2,14	0,00	0,00
d_2_7	59,89	0,00	2,44	2,71	0,55
d_2_8	51,35	0,00	1,77	1,87	0,53
d_2_11	64,79	-0,01	1,95	2,08	0,53
d_2_12	65,12	0,02	2,08	1,91	0,46
d_2_13	66,87	0,02	1,71	1,82	0,53
d_2_14	61,68	0,01	2,16	2,14	0,50
d_2_15	53,62	0,00	2,01	1,58	0,38
d_2_16	45,94	0,02	2,45	1,96	0,39
d_2_17	71,90	0,07	2,68	2,71	0,51
d_2_18	90,68	0,06	2,17	2,86	0,64
d_2_19	81,63	0,06	3,04	2,64	0,43
d_2_20	70,88	0,08	2,52	3,08	0,60
d_2_21	94,17	0,01	3,46	2,58	0,36
d_3_4	14,08	-0,01	1,34	0,42	0,09
d_3_5	24,14	0,01	1,12	1,23	0,55
d_3_6	35,55	0,01	1,78	1,11	0,28
d_3_7	49,94	0,02	2,55	2,58	0,51
d_3_11	48,05	0,02	1,65	1,92	0,58
d_3_12	48,01	0,05	1,55	1,87	0,59
d_3_13	50,62	0,05	1,10	1,78	0,72
d_3_14	48,16	0,04	1,66	2,30	0,66
d_3_15	41,49	0,03	1,31	1,59	0,59
d_3_16	34,33	0,05	1,83	1,96	0,53
d_3_17	58,80	0,09	2,18	2,68	0,60
d_3_18	73,62	0,09	1,82	2,80	0,70
d_3_19	68,46	0,09	2,50	2,66	0,53
d_3_20	61,02	0,09	2,26	2,81	0,61

Continued on next page

Response	intercept	age	σ_1	σ_2	h^2
d_3_21	80,92	0,04	3,29	2,46	0,36
d_4_5	34,79	0,01	1,16	1,25	0,53
d_4_6	45,25	0,01	1,66	1,42	0,42
d_4_7	52,00	0,02	2,29	2,37	0,52
d_4_12	55,20	0,03	1,45	1,93	0,64
d_4_13	56,04	0,04	1,36	1,73	0,62
d_4_14	50,25	0,03	1,57	2,14	0,65
d_4_15	40,69	0,02	1,47	1,50	0,51
d_4_16	32,72	0,04	1,49	2,15	0,68
d_4_17	59,69	0,08	2,24	2,55	0,56
d_4_18	78,65	0,08	1,99	2,64	0,64
d_4_19	68,65	0,08	2,42	2,62	0,54
d_4_20	56,69	0,10	2,28	2,87	0,61
d_4_21	80,55	0,03	3,13	2,53	0,39
d_5_12	43,04	0,06	2,02	1,89	0,47
d_5_13	47,60	0,06	1,65	1,68	0,51
d_5_18	72,69	0,12	2,28	2,92	0,62
d_6_12	61,55	0,06	2,67	2,07	0,38
d_6_13	66,56	0,05	2,39	1,36	0,25
d_6_18	91,08	0,12	2,71	3,12	0,57
d_7_12	30,78	0,00	1,52	1,87	0,60
d_7_13	28,25	0,01	1,83	2,02	0,55
d_7_18	51,65	0,08	1,99	2,47	0,61
d_12_13	7,52	0,01	1,31	0,80	0,27
d_12_14	19,68	0,02	1,57	1,04	0,31
d_12_15	28,93	0,04	1,19	1,71	0,67
d_12_17	26,63	0,05	1,59	1,24	0,38
d_12_18	29,41	0,08	2,08	1,60	0,37
d_12_19	35,32	0,06	1,71	1,80	0,53
d_12_20	49,25	0,07	1,54	2,14	0,66
d_12_21	47,96	0,00	3,10	1,50	0,19
d_13_14	14,60	0,02	1,16	1,07	0,46
d_13_17	19,34	0,07	1,98	1,56	0,38
d_13_18	26,40	0,08	2,51	2,40	0,48
d_13_19	28,07	0,07	2,54	1,98	0,38
d_13_20	43,33	0,08	2,53	2,18	0,43
d_13_21	41,13	0,01	3,58	1,85	0,21
d_14_18	35,79	0,08	2,44	2,35	0,48
d_17_18	31,16	0,01	1,88	1,58	0,41
d_18_19	28,26	0,02	1,88	2,03	0,54
d_18_21	30,58	0,01	1,91	2,23	0,58
d_1_10	86,31	0,00	2,12	2,64	0,61
d_2_9	65,54	-0,02	2,65	2,35	0,44
d_3_8	34,72	0,04	1,86	1,87	0,50
d_4_11	60,91	0,00	2,35	2,11	0,45
d_5_6	19,53	0,00	2,27	1,29	0,24
d_5_7	41,80	0,05	2,73	2,62	0,48
d_5_14	47,27	0,06	2,08	2,11	0,51
d_5_17	59,37	0,13	2,67	2,69	0,50

Continued on next page

Response	intercept	age	σ_1	σ_2	h^2
d_5_19	70,86	0,12	4,07	0,00	0,00
d_5_21	85,20	0,06	3,59	2,75	0,37
d_6_7	58,97	0,05	3,16	2,48	0,38
d_6_14	66,59	0,06	2,79	1,86	0,31
d_6_17	78,58	0,13	3,03	2,87	0,47
d_6_19	90,17	0,12	3,30	2,92	0,44
d_6_21	104,60	0,05	3,90	2,84	0,35
d_7_14	18,86	0,01	1,80	1,29	0,34
d_7_17	27,78	0,10	2,22	1,85	0,41
d_7_19	39,64	0,08	2,59	2,52	0,49
d_7_21	55,27	0,00	3,60	2,78	0,37
d_12_16	31,66	0,03	1,51	1,87	0,61
d_13_15	24,43	0,05	1,71	1,55	0,45
d_14_17	11,58	0,09	2,38	1,98	0,41
d_14_19	23,65	0,08	2,81	2,28	0,40
d_14_21	38,98	0,01	3,29	2,97	0,45
d_17_19	12,29	-0,01	2,13	0,67	0,09
d_17_21	27,88	-0,09	3,48	0,34	0,01
d_18_20	48,77	0,02	2,69	2,87	0,53
d_19_21	15,54	-0,07	3,24	0,10	0,00
ar_18_12_10	71,15	10,58	38,39	100,00	0,87
ar_8_7_5	455,48	1,60	41,90	49,52	0,58
ar_8_7_12	527,87	0,60	40,85	50,10	0,60
ar_8_6_5	223,06	-0,01	32,88	0,10	0,00
ar_8_9_6	338,75	-0,51	30,56	23,24	0,37
ar_11_9_10	108,45	-0,17	16,72	0,47	0,00
ar_11_8_9	101,68	-0,26	15,17	0,00	0,00
ar_11_12_10	183,00	0,21	33,96	0,00	0,00
ar_11_8_12	219,16	0,19	26,07	17,62	0,31
ar_19_18_21	219,28	-0,89	50,19	0,00	0,00
ar_19_17_18	174,71	-0,10	34,23	10,97	0,09
ar_13_18_12	95,57	0,23	18,39	14,45	0,38
ar_13_17_18	254,17	1,38	55,73	0,00	0,00
ar_13_7_12	103,92	-0,03	25,34	19,25	0,37
ar_14_13_7	122,52	0,35	14,82	13,30	0,45
ar_14_13_17	80,06	0,90	24,92	18,90	0,37
an_18_12_10_a	0,69	0,00	0,04	0,05	0,59
an_18_12_10_b	2,02	0,00	0,06	0,09	0,69
an_18_12_10_c	0,43	0,00	0,04	0,04	0,50
an_8_7_5_a	0,99	0,00	0,06	0,05	0,34
an_8_7_5_b	0,50	0,00	0,04	0,03	0,44
an_8_7_5_c	1,65	0,00	0,06	0,05	0,34
an_8_7_12_a	0,66	0,00	0,05	0,04	0,45
an_8_7_12_b	0,75	0,00	0,07	0,06	0,45
an_8_7_12_c	1,74	0,00	0,12	0,00	0,00
an_8_6_5_a	0,54	0,00	0,07	0,00	0,00
an_8_6_5_b	0,71	0,00	0,05	0,05	0,42
an_8_6_5_c	1,89	0,00	0,07	0,05	0,34
an_8_9_6_a	1,46	0,00	0,08	0,04	0,24

Continued on next page

Response	intercept	age	σ_1	σ_2	h^2
an_8_9_6_b	1,17	0,00	0,08	0,04	0,17
an_8_9_6_c	0,52	0,00	0,04	0,03	0,28
an_11_9_10_a	1,25	0,00	0,10	0,08	0,35
an_11_9_10_b	0,98	0,00	0,09	0,07	0,34
an_11_9_10_c	0,91	0,00	0,09	0,03	0,11
an_11_8_9_a	1,47	0,00	0,13	0,00	0,00
an_11_8_9_b	0,86	0,00	0,10	0,03	0,07
an_11_8_9_c	0,82	0,00	0,11	0,03	0,07
an_11_12_10_a	2,36	0,00	0,11	0,00	0,00
an_11_12_10_b	0,25	0,00	0,04	0,02	0,21
an_11_12_10_c	0,53	0,00	0,07	0,05	0,34
an_11_8_12_a	1,46	0,00	0,09	0,04	0,16
an_11_8_12_b	1,26	0,00	0,08	0,04	0,23
an_11_8_12_c	0,42	0,00	0,04	0,02	0,20
an_19_18_21_a	1,46	0,00	0,11	0,07	0,28
an_19_18_21_b	0,52	0,00	0,10	0,02	0,05
an_19_18_21_c	1,16	0,00	0,15	0,01	0,00
an_19_17_18_a	1,61	0,00	0,10	0,07	0,28
an_19_17_18_b	1,13	0,00	0,07	0,08	0,55
an_19_17_18_c	0,41	0,00	0,08	0,03	0,17
an_13_18_12_a	1,89	0,00	0,33	0,13	0,14
an_13_18_12_b	0,24	0,00	0,03	0,03	0,46
an_13_18_12_c	1,01	0,00	0,32	0,13	0,15
an_13_17_18_a	1,44	0,00	0,12	0,11	0,48
an_13_17_18_b	1,02	0,00	0,08	0,09	0,55
an_13_17_18_c	0,68	0,00	0,06	0,04	0,33
an_13_7_12_a	1,80	0,00	0,33	0,23	0,32
an_13_7_12_b	0,24	0,00	0,06	0,04	0,31
an_13_7_12_c	1,09	0,00	0,35	0,03	0,01
an_14_13_7_a	2,01	0,00	0,17	0,11	0,30
an_14_13_7_b	0,66	0,00	0,12	0,06	0,22
an_14_13_7_c	0,48	0,00	0,10	0,07	0,28
an_14_13_17_a	1,63	0,00	0,19	0,00	0,00
an_14_13_17_b	0,66	0,00	0,11	0,09	0,44
an_14_13_17_c	0,85	0,00	0,09	0,07	0,41
